# Effects of Roxadustat on the Anemia and Iron Metabolism of Patients Undergoing Peritoneal Dialysis

**DOI:** 10.3389/fmed.2021.667117

**Published:** 2021-07-07

**Authors:** Keiji Hirai, Hiroaki Nonaka, Moeka Ueda, Junki Morino, Shohei Kaneko, Saori Minato, Yuko Mutsuyoshi, Katsunori Yanai, Hiroki Ishii, Momoko Matsuyama, Taisuke Kitano, Akinori Aomatsu, Haruhisa Miyazawa, Kiyonori Ito, Yuichiro Ueda, Susumu Ookawara, Yoshiyuki Morishita

**Affiliations:** Division of Nephrology, First Department of Integrated Medicine, Saitama Medical Center, Jichi Medical University, Saitama, Japan

**Keywords:** roxadustat, anemia, peritoneal dialysis, erythropoiesis-stimulating agent, iron metabolism

## Abstract

**Background:** We investigated the effects of roxadustat on the anemia, iron metabolism, peritoneal membrane function, and residual renal function; and determined the factors associated with the administration of roxadustat in patients who were undergoing peritoneal dialysis.

**Methods:** We retrospectively analyzed the changes in hemoglobin, serum ferritin, transferrin saturation (TSAT), 4-h dialysate/plasma creatinine, and renal weekly urea clearance over the 24 weeks following the change from an erythropoiesis-stimulating agent (ESA) to roxadustat in 16 patients who were undergoing peritoneal dialysis and had anemia (Roxadustat group). Twenty-three peritoneal dialysis patients who had anemia and continued ESA served as a control group (ESA group).

**Results:** There were no significant differences in hemoglobin, serum ferritin, TSAT, 4-h dialysate/plasma creatinine, or renal weekly urea clearance between the two groups at baseline. The hemoglobin concentration was significantly higher in the Roxadustat group than in the ESA group after 24 weeks (11.6 ± 1.0 g/dL vs. 10.3 ± 1.1 g/dL, *p* < 0.05), whereas the ferritin concentration and TSAT were significantly lower (139.5 ± 102.0 ng/mL vs. 209.2 ± 113.1 ng/mL, *p* < 0.05; and 28.1 ± 11.5% vs. 44.8 ± 10.4%, *p* < 0.05, respectively). The changes in 4-h dialysate/plasma creatinine and renal weekly urea clearance did not differ between the two groups. Linear regression analysis revealed that the serum potassium concentration correlated with the dose of roxadustat at 24 weeks (standard coefficient = 0.580, *p* = 0.019).

**Conclusion:** Roxadustat may improve the anemia and reduce the serum ferritin and TSAT of the peritoneal dialysis patients after they were switched from an ESA, without association with peritoneal membrane function or residual renal function.

## Introduction

Anemia is a frequently observed complication in patients undergoing peritoneal dialysis and is associated with lower residual renal function, poorer quality of life, and a higher risk of mortality (1–3). Therefore, optimal treatment of anemia is important to maintain residual renal function and improve the quality of life and prognosis of peritoneal dialysis patients (4).

Erythropoiesis-stimulating agents (ESAs) have been widely used to maintain appropriate hemoglobin levels in peritoneal dialysis patients (5). However, they require subcutaneous administration, which is associated with pain for patients and the risk of needle-stick injury for nurses.

Roxadustat is a novel orally administered hypoxia-inducible factor prolyl hydroxylase inhibitor that increases endogenous erythropoietin concentration through the stabilization of hypoxia inducible factor, which is achieved by inhibiting the activity of its pan-prolyl hydroxylase domain (6), and it has recently been approved for the treatment of anemia in patients with chronic kidney disease (CKD) (7). A phase III clinical trial showed that roxadustat increased the hemoglobin concentration and reduced the serum ferritin concentration and TSAT after patients undergoing peritoneal dialysis were switched from an ESA (8). However, real-world data regarding the effects of roxadustat on peritoneal membrane function and residual renal function are scarce. Furthermore, the factors associated with the dose of roxadustat required to maintain the hemoglobin level have yet to be determined. Therefore, in the present study, we investigated the effects of roxadustat on peritoneal membrane function and residual renal function, and the factors associated with the dose of roxadustat, as well as the effects of roxadustat on the anemia and iron metabolism of patients who were undergoing peritoneal dialysis and being administered ESA in a real-world clinical setting.

## Materials and Methods

### Ethics Approval

The study was approved by the ethics committee of Saitama Medical Center, Jichi Medical University (RIN 15-33), and was conducted according to the principles contained within the Declaration of Helsinki. Informed consent was not applicable because of the retrospective nature of the study. Information regarding this study was displayed on notice boards in the patient waiting rooms of our institution to inform all of the patients of their right to opt out.

### Participants

We analyzed the data from patients who had been treated at the Saitama Medical Center, Jichi Medical University, between 2019 and 2020. The inclusion criteria were: (i) age >20 years, (ii) CKD stage G5D; (iii) regular peritoneal dialysis, and (iv) administration of an ESA for ≥48 weeks or administration of roxadustat for ≥24 weeks after being administered an ESA for ≥24 weeks. The exclusion criteria were: (i) hemodialysis, (ii) renal transplantation, and (iii) poor compliance with the roxadustat treatment.

### Study Design

**Figure 1:**
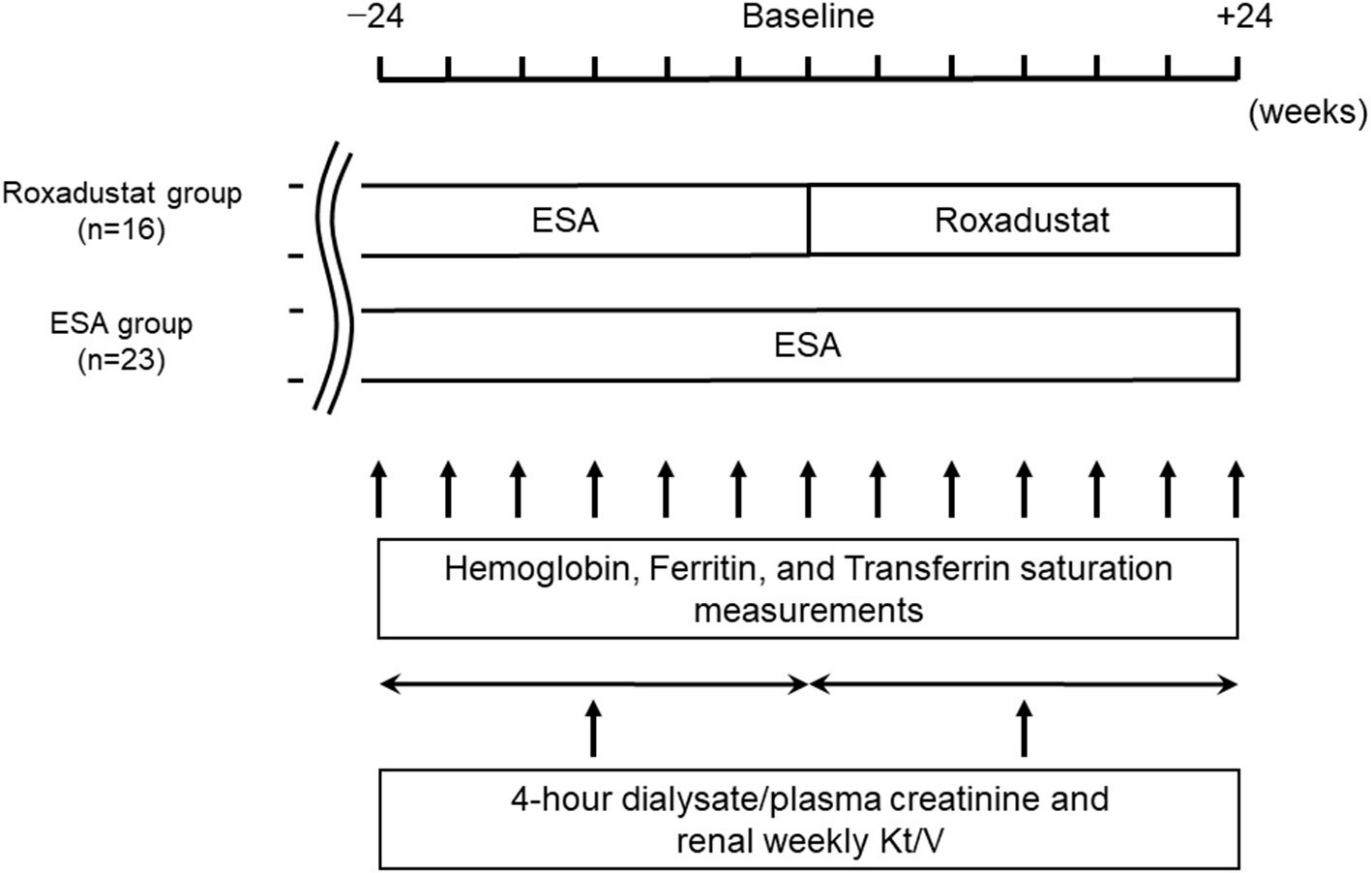
Study design. ESA, erythropoiesis-stimulating agent.

We performed a retrospective comparative study of 39 patients. The study design is illustrated in Figure 1. The demographic and clinical data for each participant were obtained from their medical records. We allocated 16 patients who had been taking roxadustat for ≥24 weeks after being administered an ESA for ≥24 weeks to the Roxadustat group and 23 patients who had been administered an ESA for ≥48 weeks to the ESA (control) group. The baseline for each participant in the ESA group was between January 1, 2020 and April 1, 2020, during which time the participants in the Roxadustat group started taking roxadustat. Roxadustat was orally administered three times per week, at bedtime, and ESAs were subcutaneously administered once per month on the day of a hospital visit. The changes in hemoglobin, serum ferritin, and transferrin saturation (TSAT) between baseline and 24 weeks later were evaluated in each group. Four-hour dialysate/plasma creatinine and renal weekly urea clearance (Kt/V) were evaluated within 24 weeks before and after baseline in each group. The factors associated with the dose of roxadustat at +24 weeks were identified using multiple linear regression analysis.

### Laboratory Methods

Blood parameters and peritoneal dialysis fluid parameters were measured by the Department of Clinical Laboratory, Saitama Medical Center. The erythropoietin resistance index was calculated as the mean weekly dose of epoetin (IU)/body mass (kg)/hemoglobin (g/dL) (9). For the conversion of ESA doses to doses of epoetin, ratios of 200:1 and 225:1 were used for darbepoetin alfa and epoetin beta pegol, respectively (10). The total weekly Kt/V was measured by calculating the sum of the residual renal and peritoneal clearances of urea and converting this to a weekly value (11).

### Statistics

Statistical analyses were performed using JMP v11 (SAS Institute, Cary, North Carolina, USA). Continuous variables are expressed as mean ± standard deviation when they were normally distributed and as median and interquartile range when non-normally distributed. Categorical variables are expressed as numbers and percentages. Comparisons of clinical data between two groups were performed using Student's *t*-test for normally-distributed data and the Mann-Whitney *U*-test for non-normally-distributed data. Comparisons of component ratios between two groups were performed using Fisher's exact-test. Comparisons of serial measurements within each group were performed using repeated-measures analysis of variance, followed by Tukey's test. Parameters that significantly correlated with the dose of roxadustat at +24 weeks were included in multiple linear regression analysis to identify those that independently correlated with the doses of these drugs at +24 weeks. *P* < 0.05 was considered to represent statistical significance.

## Results

### Patient Characteristics

**Figure 2:**
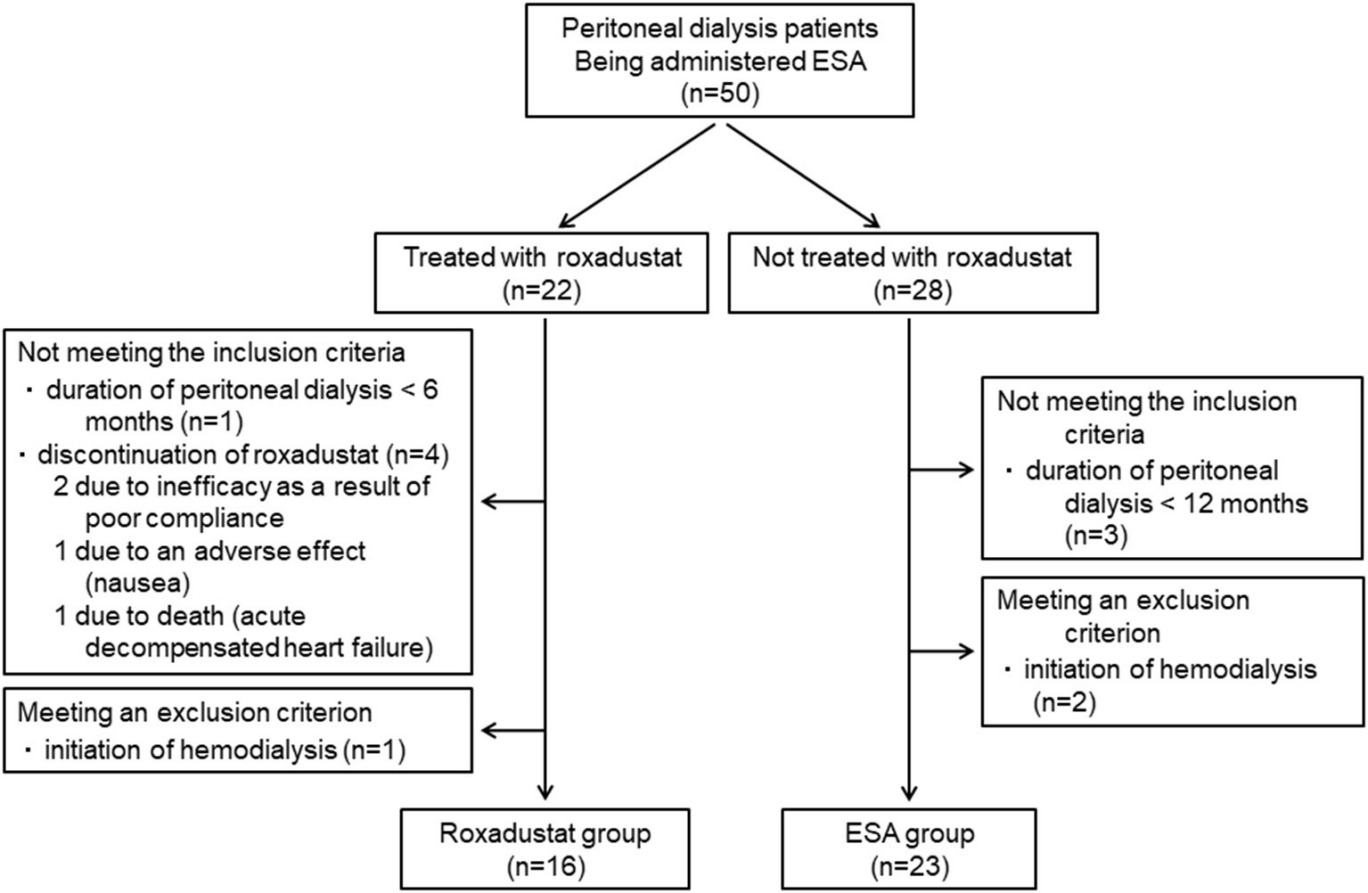
Patient flow diagram. ESA, erythropoiesis-stimulating agent.

A total of 50 patients who were undergoing peritoneal dialysis and being administered an ESA were identified. Forty-three of these patients were being administered an ESA before the induction of peritoneal dialysis. Twenty-two of these patients were treated with roxadustat in place of ESA and 28 were treated with an ESA alone. The reasons for changing the ESA to roxadustat were insufficient efficacy of the former in two patients and injection pain in 20 patients. Five patients who were treated with roxadustat did not meet the inclusion criteria and one patient met an exclusion criterion, which meant that 16 patients comprised the Roxadustat group. Three patients who were treated with an ESA alone did not meet the inclusion criteria and two patients met an exclusion criterion, resulting in the inclusion of 23 patients in the ESA group (Figure 2). Therefore, data from 39 patients (23 men and 16 women, mean age 60.4 ± 13.9 years, mean body mass index 22.9 ± 4.2 kg/m^2^, mean duration of peritoneal dialysis 36.8 ± 36.7 months) were analyzed.

Their mean hemoglobin and ferritin concentrations and TSAT were 10.7 ± 1.3 g/dL, 166.1 ± 107.2 ng/mL, and 39.3 ± 12.3%, respectively. Twenty-two patients had been treated with darbepoetin alfa and 17 with epoetin beta pegol once per month. The mean ESA dose was 5,231 ± 2,319 IU/week and the mean erythropoietin resistance index was 8.7 ± 4.4. Thirty-one patients (79.5%) were undergoing continuous ambulatory peritoneal dialysis (CAPD), 24 patients (61.5%) were undergoing automated peritoneal dialysis (APD), and 16 patients (41.0%) were undergoing a combination of CAPD and APD.

The percentages of the patients in which each peritoneal dialysis solution was used were as follows: icodextrin solution, 64.1%; lactate-buffered solution, 20.5%; and bicarbonate-buffered solution, 79.5%. The mean total weekly Kt/V was 1.71 ± 0.40, the renal weekly Kt/V was 0.58 ± 0.44, the weekly peritoneal Kt/V was 1.13 ± 0.43, and the 4-h dialysate/plasma creatinine was 0.65 ± 0.11. The mean systolic and diastolic blood pressure were 141.8 ± 18.3 mmHg and 81.2 ± 15.7 mmHg, respectively. The percentages of the patients with histories of diabetes mellitus, myocardial infarction, and stroke were 30.8, 15.4, and 7.7%, respectively. The proportions of participants taking each medication were: calcium-containing phosphate binders, 43.6%; calcium-free phosphate binders, 79.5%; vitamin D analogs, 64.1%; calcimimetics, 20.5%; iron supplements, 51.3%; zinc supplements, 12.8%; and carnitine supplements, 10.3%. The administered doses of each iron supplement were: sodium ferrous citrate 50 mg/day (three patients), ferric citrate hydrate 500 mg/day (three patients), 750 mg/day (five patients), and 1,500 mg/day (five patients); and sucroferric oxyhydroxide 500 mg/day (two patients) and 750 mg/day (two patients). The doses of the iron supplements were not changed during the study period. The values of the other clinical parameters were as follows: albumin, 3.4 ± 0.5 g/dL; blood urea nitrogen, 58.4 ± 12.6 mg/dL; serum creatinine, 10.8 ± 2.9 mg/dL; uric acid, 6.0 ± 1.1 mg/dL; sodium, 137.9 ± 3.3 mEq/L; potassium, 4.3 ± 0.7 mEq/L; chloride, 100.0 ± 5.1 mEq/L; total calcium, 8.4 ± 0.7 mg/dL; phosphorus, 5.7 ± 1.2 mg/dL; magnesium, 2.1 ± 0.4 mg/dL; intact parathyroid hormone, 296.6 ± 192.4 pg/mL; C-reactive protein, 0.19 ± 0.25 mg/dL; β2 microglobulin, 26.7 ± 9.4 mg/L. The baseline patient characteristics of and the medications being administered to the two groups are summarized in Table 1. There were no significant differences in the clinical parameters between the two groups, except for body mass index and the proportion of patients undergoing CAPD.

**Table 1:** Baseline patient characteristics.

			**Roxadustat group (*n* = 16)**	**ESA group (*n* = 23)**	***p*-value**
Age (years)			58.0 ± 13.8	62.1 ± 13.9	0.32
Male sex [number (%)]			7 (43.8)	16 (69.6)	0.19
Body mass index (kg/m^2^)			20.7 ± 3.3	24.5 ± 4.1	0.005^*^
Systolic blood pressure (mmHg)			136.8 ± 15.3	145.2 ± 19.7	0.18
Diastolic blood pressure (mmHg)			81.4 ± 13.4	81.1 ± 17.4	0.90
Duration of peritoneal dialysis (months)			40.8 (18.0–95.4)	23.0 (10.0–56.5)	0.27
Peritoneal dialysis modality [number (%)]		CAPD	10 (62.5)	21 (91.3)	0.045^*^
		APD	12 (75.0)	12 (52.2)	0.19
		CAPD and APD	6 (37.5)	10 (43.5)	0.75
Peritoneal dialysis solution [number (%)]		Icodextrin solution	8 (50.0)	17 (73.9)	0.18
		Lactate-buffered solution	3 (18.8)	5 (21.7)	0.82
		Bicarbonate-buffered solutio	13 (81.3)	18 (78.3)	0.82
Total weekly Kt/V			1.78 ± 0.38	1.68 ± 0.45	0.16
Renal weekly Kt/V			0.60 ± 0.37	0.57 ± 0.49	0.23
Peritoneal weekly Kt/V			1.14 ± 0.52	1.12 ± 0.37	0.91
4-hour dialysate/plasma creatinine			0.64 ± 0.09	0.65 ± 0.13	0.64
Diabetes mellitus [number (%)]			4 (25.0)	8 (34.8)	0.73
Previous myocardial infarction [number (%)]			2 (12.5)	4 (17.4)	0.67
Previous stroke [number (%)]			2 (12.5)	1 (4.3)	0.56
Calcium-containing phosphate binder use [number (%)]			5 (31.3)	12 (52.2)	0.33
Calcium-free phosphate binder use [number (%)]			12 (75.0)	19 (82.6)	0.69
Vitamin D analog use [number (%)]			11 (68.8)	14 (60.9)	0.74
Calcimimetic use [number (%)]			2 (12.5)	6 (26.1)	0.43
Iron supplement use [number (%)]	Sodium ferrous citrate	50 mg/day	2 (12.5)	1 (4.3)	0.30
	Ferric citrate hydrate	500 mg/day	0 (0.0)	3 (13.0)	
		750 mg/day	4 (25.0)	1 (4.3)	
		1,500 mg/day	1 (6.3)	4 (17.4)	
	Sucroferric oxyhydroxide	500 mg/day	1 (6.3)	1 (4.3)	
		750 mg/day	1 (6.3)	1 (4.3)	
Zinc supplement use [number (%)]			3 (18.8)	2 (8.7)	0.63
Carnitine supplement use [number (%)]			3 (18.8)	1 (4.3)	0.29
Albumin (g/dL)			3.4 ± 0.3	3.4 ± 0.6	0.72
Hemoglobin (g/dL)			10.7 ± 1.2	10.5 ± 1.3	0.61
Blood urea nitrogen (mg/dL)			56.3 ± 10.0	59.9 ± 14.1	0.58
Serum creatinine (mg/dL)			10.2 ± 3.0	11.2 ± 2.8	0.41
Uric acid (mg/dL)			6.1 ± 1.1	5.9 ± 1.1	0.63
Sodium (mEq/L)			138.5 ± 2.5	137.5 ± 3.8	0.34
Potassium (mEq/L)			4.3 ± 0.7	4.3 ± 0.6	0.75
Chloride (mEq/L)			100.7 ± 4.6	99.6 ± 5.5	0.41
Total calcium (mg/dL)			8.5 ± 0.7	8.4 ± 0.8	0.23
Phosphorus (mg/dL)			5.6 ± 1.4	5.7 ± 1.0	0.67
Magnesium (mg/dL)			2.0 ± 0.4	2.1 ± 0.4	0.46
Intact-parathyroid hormone (pg/mL)			229.4 ± 133.6	343.4 ± 215.0	0.09
Ferritin (ng/mL)			145.1 ± 129.6	180.7 ± 88.7	0.07
Transferrin saturation (%)			32.6 ± 9.2	40.9 ± 12.9	0.31
C-reactive protein (mg/dL)			0.22 (0.06–0.29)	0.08 (0.04–0.35)	0.57
β2 microglobulin (mg/L)			25.7 ± 11.2	27.5 ± 8.0	0.36
Erythropoiesis-stimulating agent [number (%)]		Darbepoetin alfa	9 (56.3)	13 (56.5)	1.00
		Epoetin beta pegol	7 (43.8)	10 (43.5)	
Erythropoiesis-stimulating agent dose (IU/week)			4,813 ± 2,272	5,478 ± 2,318	0.34
Erythropoietin resistance index [IU/week/kg/(g/dL)]			9.0 ± 5.0	8.4 ± 4.0	0.82

*APD, automated peritoneal dialysis; CAPD, continuous ambulatory peritoneal dialysis; ESA, erythropoiesis-stimulating agent; IU, international units; Kt/V, urea clearance. Data are presented as means ± standard deviations, medians (interquartile ranges), or numbers (%)*.

* *P-values are statistically significant*.

### Changes in ESA and Roxadustat Doses and Erythropoietin Resistance Index

**Figure 3:**
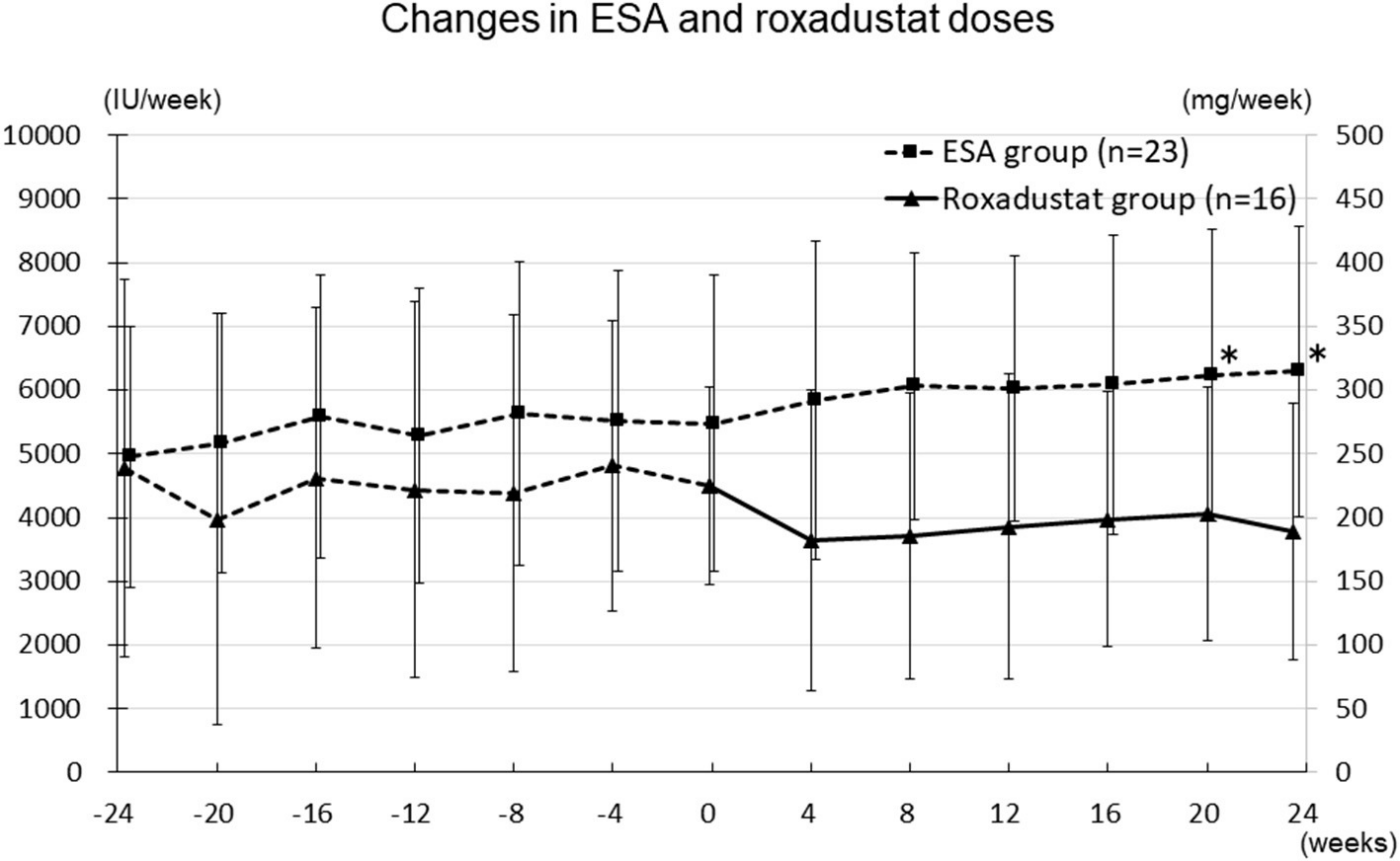
Changes in the ESA (IU/week) and roxadustat (mg/week) doses administered during the study. ESA, erythropoiesis-stimulating agent; IU, international units; *p < 0.05 vs. −24 weeks.

**Figure 4:**
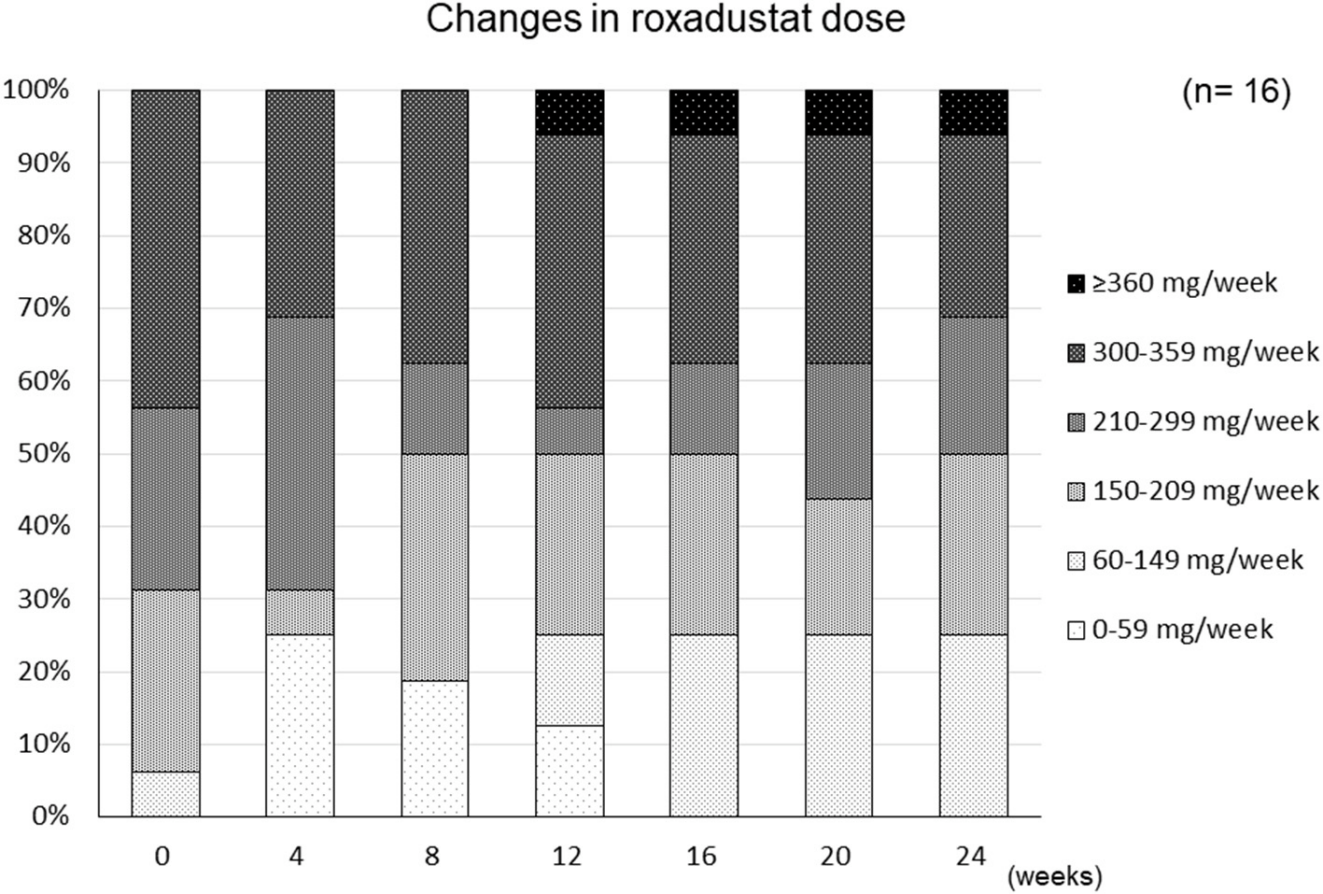
Changes in the distribution of the roxadustat dose administered to participants during the study period.

**Figure 5:**
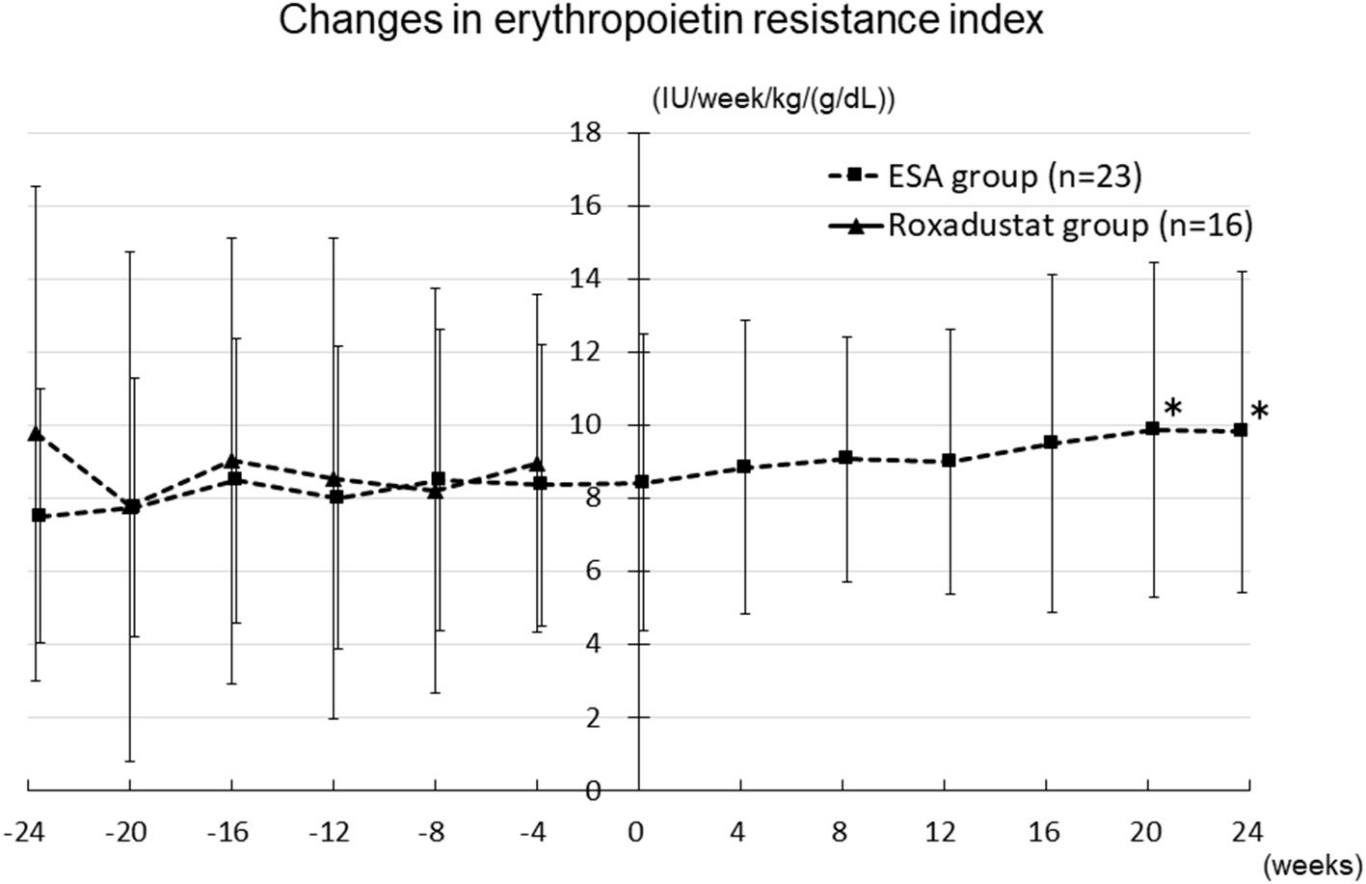
Changes in the erythropoietin resistance index in the Roxadustat and ESA groups. ESA, erythropoiesis-stimulating agent; IU, international units; *p < 0.05 vs. −24 weeks.

The changes in ESA and roxadustat doses in each group are shown in Figure 3. The distribution of roxadustat dose after baseline in participants in the Roxadustat group is shown in Figure 4. The dose of ESA significantly increased from 4,957 ± 2,050 IU/week at −24 weeks to 6,239 ± 2,286 IU/week at +20 weeks (*p* < 0.05) and 6,293 ± 2,270 IU/week at +24 weeks (*p* < 0.05) in the ESA group. The dose of ESA did not change before baseline in the Roxadustat group, nor did the dose of roxadustat change after baseline in the Roxadustat group. The changes in erythropoietin resistance index in each group are shown in Figure 5. The erythropoietin resistance index significantly increased from 7.5 ± 3.5 IU/week/kg/(g/dL) at −24 weeks to 9.9 ± 4.6 IU/week/kg/(g/dL) at +20 weeks (*p* < 0.05) and 9.8 ± 4.4 IU/week/kg/(g/dL) at +24 weeks (*p* < 0.05) in the ESA group, but did not change prior to baseline in the Roxadustat group.

### Factors Associated With the Dose of Roxadustat at +24 Weeks

Simple linear regression analyses revealed that the dose of roxadustat at +24 weeks significantly correlated with potassium concentration only (standard coefficient = 0.580, *p* = 0.019) (Table 2).

**Table 2:** Simple linear regression analyses of the variables correlated with the dose of roxadustat at +24 weeks.

**Variables**	**Simple linear regression analysis**	
	**Standard coefficient**	***P-value***
Age (years)	−0.367	0.16
Male sex (yes vs. no)	−0.123	0.65
Body mass index (kg/m^2^)	0.412	0.11
Systolic blood pressure (mmHg)	−0.068	0.80
Diastolic blood pressure (mmHg)	0.132	0.63
Duration of peritoneal dialysis (months)	−0.151	0.58
CAPD (yes vs. no)	−0.124	0.65
APD (yes vs. no)	0.277	0.30
CAPD and APD (yes vs. no)	0.124	0.65
Icodextrin solution (yes vs. no)	−0.096	0.72
Lactate-buffered solution (yes vs. no)	−0.095	0.73
Bicarbonate-buffered solution (yes vs. no)	0.095	0.73
Total weekly Kt/V	−0.134	0.62
Renal weekly Kt/V	−0.204	0.45
Peritoneal weekly Kt/V	0.050	0.85
4-h dialysate/plasma creatinine	0.004	0.99
Diabetes mellitus (yes vs. no)	−0.277	0.30
Previous myocardial infarction (yes vs. no)	−0.327	0.22
Previous stroke (yes vs. no)	−0.327	0.22
Calcium-containing phosphate binder use (yes vs. no)	0.184	0.50
Calcium-free phosphate binder use (yes vs. no)	0.410	0.11
Vitamin D analog use (yes vs. no)	−0.391	0.13
Calcimimetic use (yes vs. no)	0.254	0.34
Iron supplement use (yes vs. no)	−0.070	0.80
Zinc supplement use (yes vs. no)	0.003	0.99
Carnitine supplement use (yes vs. no)	0.397	0.13
Albumin (g/dL)	0.050	0.85
Hemoglobin (g/dL)	−0.303	0.25
Blood urea nitrogen (mg/dL)	0.110	0.68
Serum creatinine (mg/dL)	0.385	0.14
Uric acid (mg/dL)	−0.213	0.43
Sodium (mEq/L)	−0.162	0.55
Potassium (mEq/L)	0.580	0.019^*^
Chloride (mEq/L)	−0.311	0.24
Total calcium (mg/dL)	0.116	0.67
Phosphorus (mg/dL)	−0.024	0.93
Magnesium (mg/dL)	0.106	0.70
Intact-parathyroid hormone (pg/mL)	0.004	0.99
Ferritin (ng/mL)	0.121	0.65
Transferrin saturation (%)	0.358	0.17
C-reactive protein (mg/dL)	0.098	0.72
β2 microglobulin (mg/L)	0.282	0.29
Erythropoiesis-stimulating agent (epoetin beta pegol vs. darbepoetin alfa)	0.264	0.32
Baseline erythropoiesis-stimulating agent dose (IU/week)	0.318	0.23
Baseline erythropoietin resistance index [IU/week/kg/(g/dL)]	0.115	0.67

*APD, automated peritoneal dialysis; CAPD, continuous ambulatory peritoneal dialysis; IU, international units; Kt/V, urea clearance*.

* *P-value is statistically significant*.

### Effect of Roxadustat on Anemia

**Figure 6:**
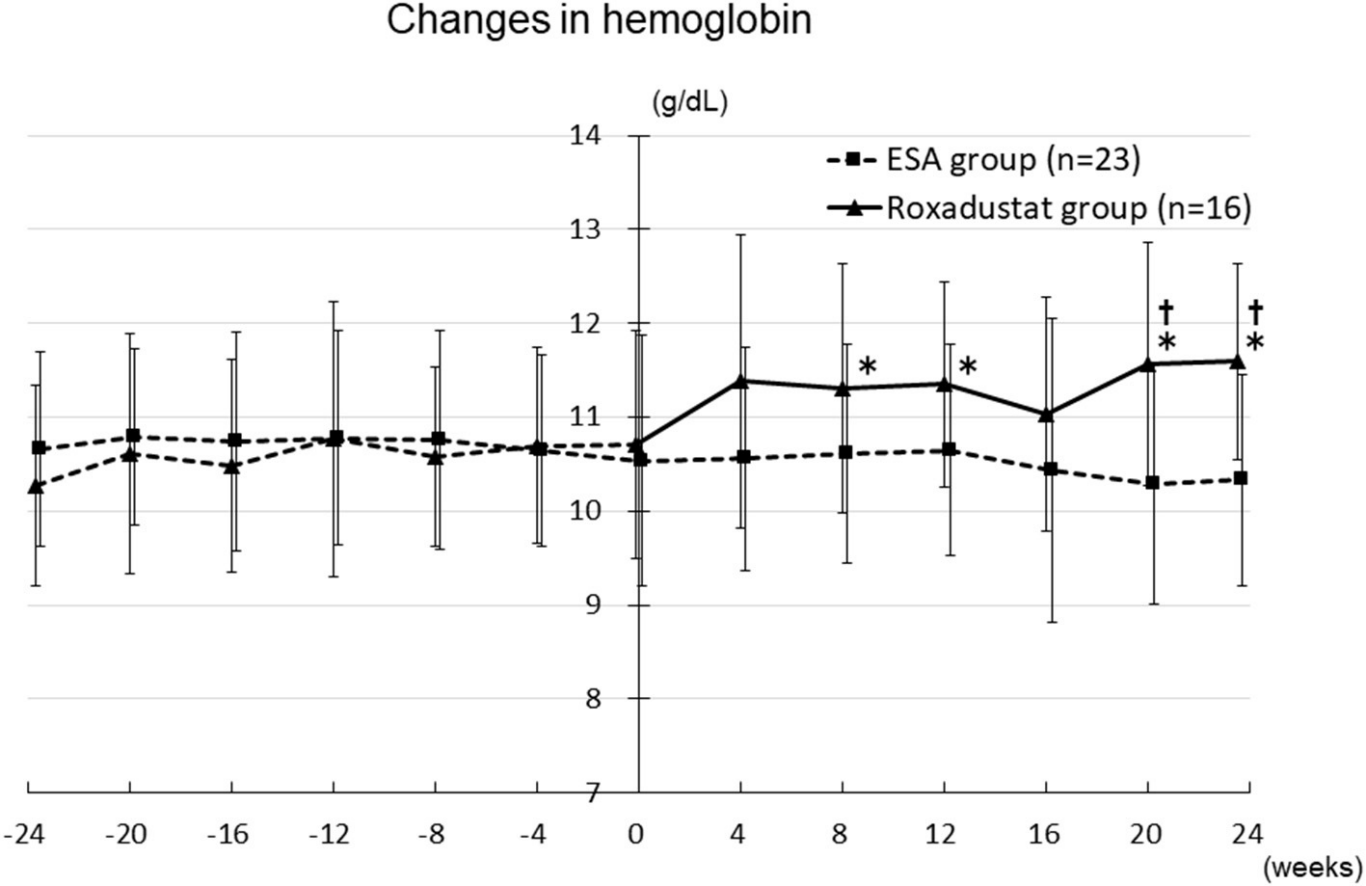
Changes in the hemoglobin concentration in the Roxadustat and ESA groups. ESA, erythropoiesis-stimulating agent; *p < 0.05 vs. −24 weeks; †*p* < 0.05 vs. the ESA group.

The hemoglobin concentration significantly increased from 10.3 ± 1.1 g/dL at −24 weeks to 11.3 ± 1.3 g/dL at 8 weeks (*p* < 0.05), 11.4 ± 1.1 g/dL at 12 weeks (*p* < 0.05), 11.6 ± 1.3 g/dL at 20 weeks (*p* < 0.05), and 11.6 ± 1.0 g/dL at 24 weeks (*p* < 0.05) in the Roxadustat group. In contrast, it did not significantly change during the study period in the ESA group. In addition, the hemoglobin concentration was significantly higher in the Roxadustat group than in the ESA group at 20 weeks (11.6 ± 1.3 g/dL vs. 10.3 ± 1.3 g/dL, *p* < 0.05) and 24 weeks (11.6 ± 1.0 g/dL vs. 10.3 ± 1.1 g/dL, *p* < 0.05) (Figure 6).

### Effects of Roxadustat on Iron Metabolism

**Figure 7:**
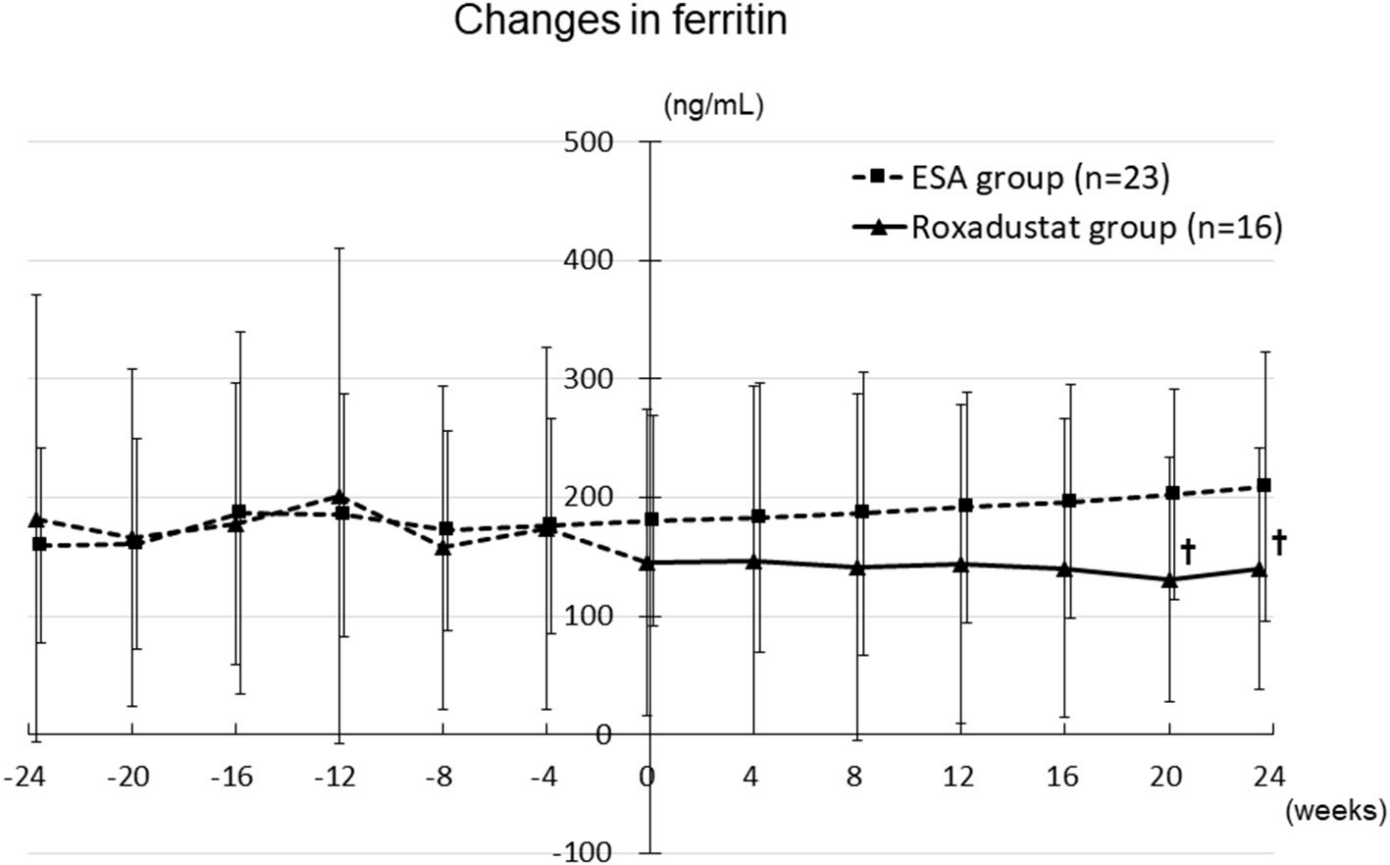
Changes in the serum ferritin concentration in the Roxadustat and ESA groups. ESA, erythropoiesis-stimulating agent; †*p* < 0.05 vs. the ESA group.

The serum ferritin concentration did not significantly change during the study period in either group, although it tended to decrease in the Roxadustat group and to increase in the ESA group. The ferritin concentration was significantly lower in the Roxadustat group than in the ESA group at 20 weeks (130.9 ± 103.5 ng/mL vs. 202.6 ± 89.2 ng/mL, *p* < 0.05) and 24 weeks (139.5 ± 102.0 ng/mL vs. 209.2 ± 113.1 ng/mL, *p* < 0.05) (Figure 7).

**Figure 8:**
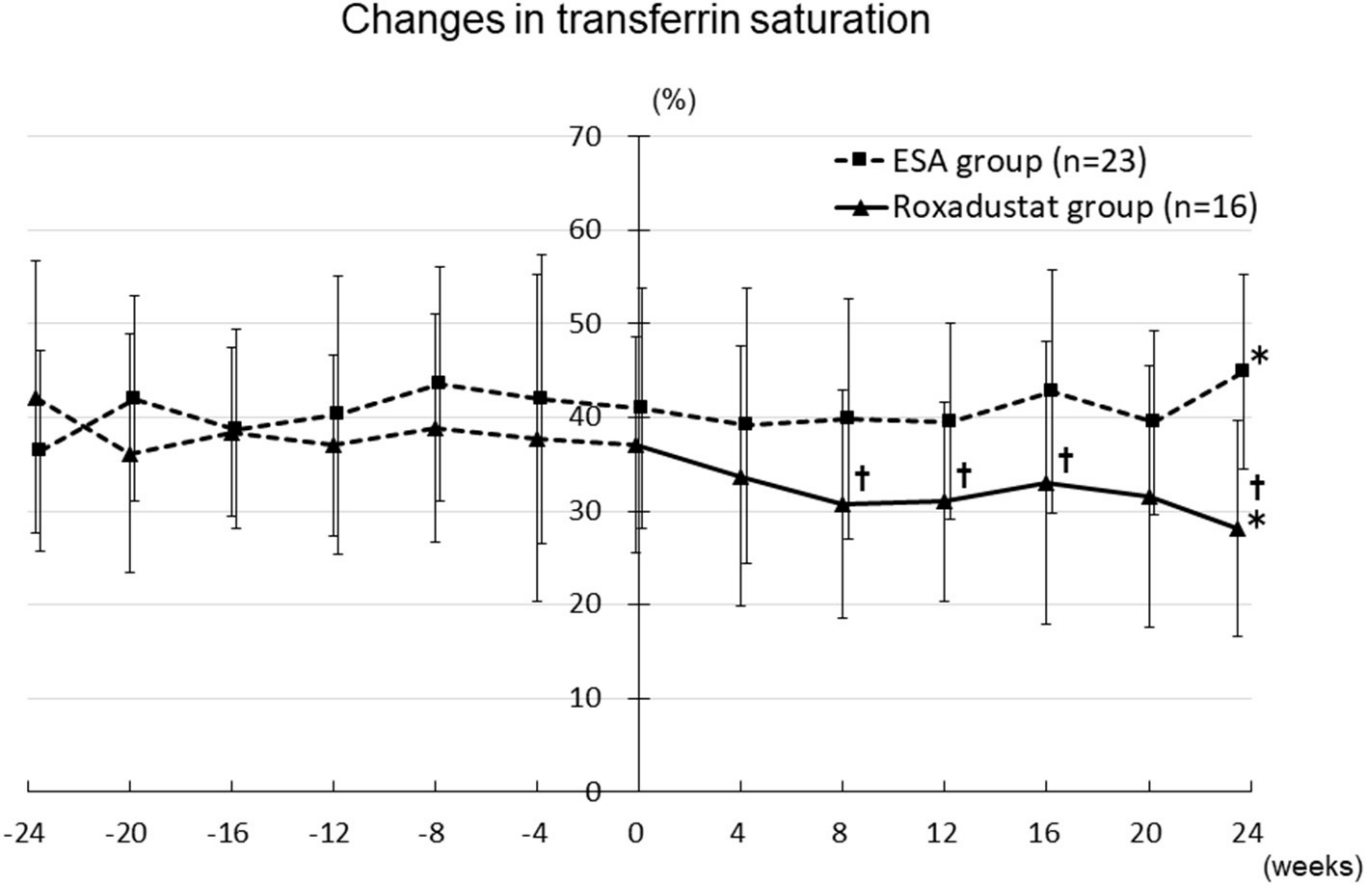
Changes in transferrin saturation in the Roxadustat and ESA groups. ESA, erythropoiesis-stimulating agent; *p < 0.05 vs. −24 weeks; †*p* < 0.05 vs. the ESA group.

The TSAT significantly decreased from 42.1 ± 14.6% at −24 weeks to 28.1 ± 11.5% at 24 weeks (*p* < 0.05) in the Roxadustat group, but significantly increased from 36.4 ± 10.7% at −24 weeks to 44.8 ± 10.4% at 24 weeks (*p* < 0.05) in the ESA group. The TSAT was significantly lower in the Roxadustat group than in the ESA group at 8 weeks (30.7 ± 12.1% vs. 39.9 ± 12.9%, *p* < 0.05), 12 weeks (31.0 ± 10.7% vs. 39.5 ± 10.5%, *p* < 0.05), 16 weeks (33.0 ± 15.2% vs. 42.7 ± 12.9%, *p* < 0.05), and 24 weeks (28.1 ± 11.5% vs. 44.8 ± 10.4%, *p* < 0.05) (Figure 8).

### Effects of Roxadustat on Peritoneal Membrane Function and Residual Renal Function

**Figure 9:**
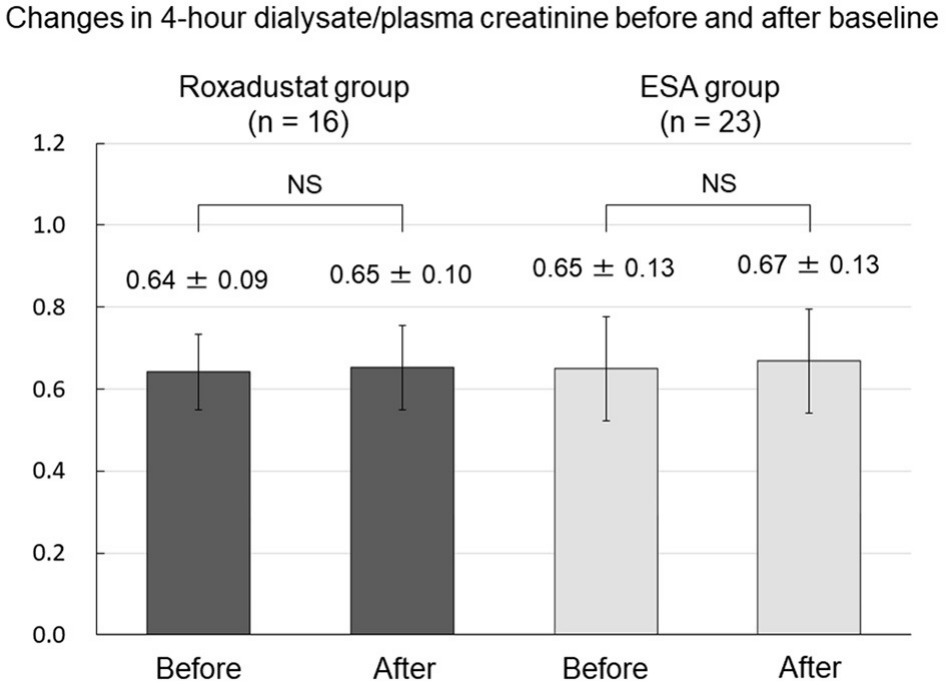
Changes in 4-h dialysate/plasma creatinine before and after baseline in the Roxadustat and ESA groups. ESA, erythropoiesis-stimulating agent; NS, not significant.

**Figure 10:**
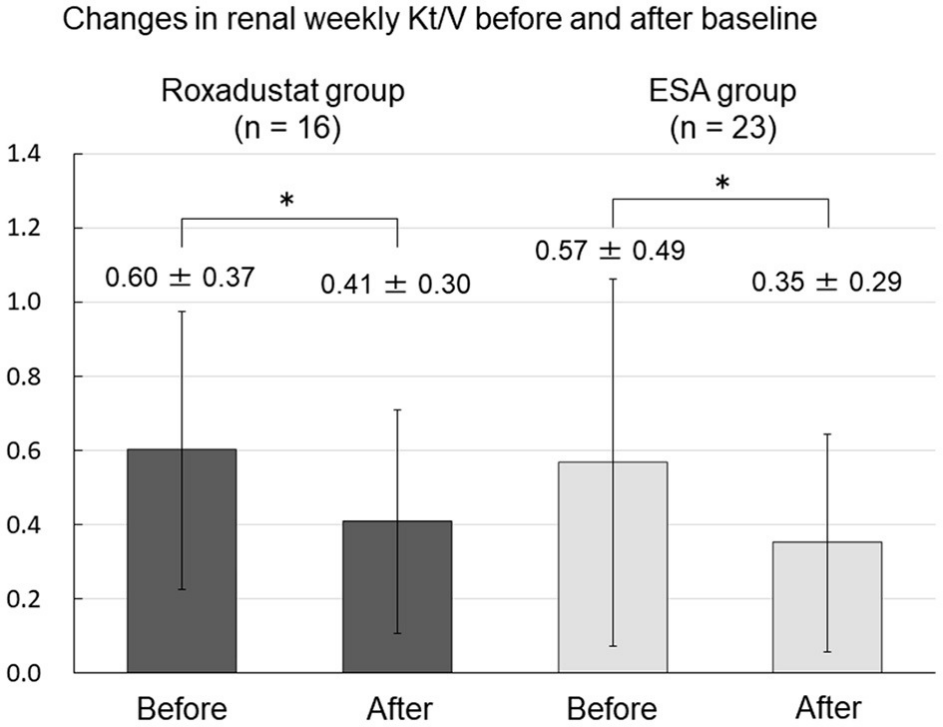
Changes in renal weekly Kt/V before and after baseline in the Roxadustat and ESA groups. ESA, erythropoiesis-stimulating agent; Kt/V, urea clearance; *p < 0.01.

There was no change in 4-h dialysate/plasma creatinine between before and after baseline in either the Roxadustat or ESA groups (Figure 9). Renal weekly Kt/V significantly decreased from 0.60 ± 0.37 and 0.57 ± 0.49 before baseline to 0.41 ± 0.30 and 0.35 ± 0.29 after baseline in both the Roxadustat and ESA groups (both *p* < 0.01) (Figure 10).

### Changes in Other Clinical Parameters and Adverse Effects

**Figure 11:**
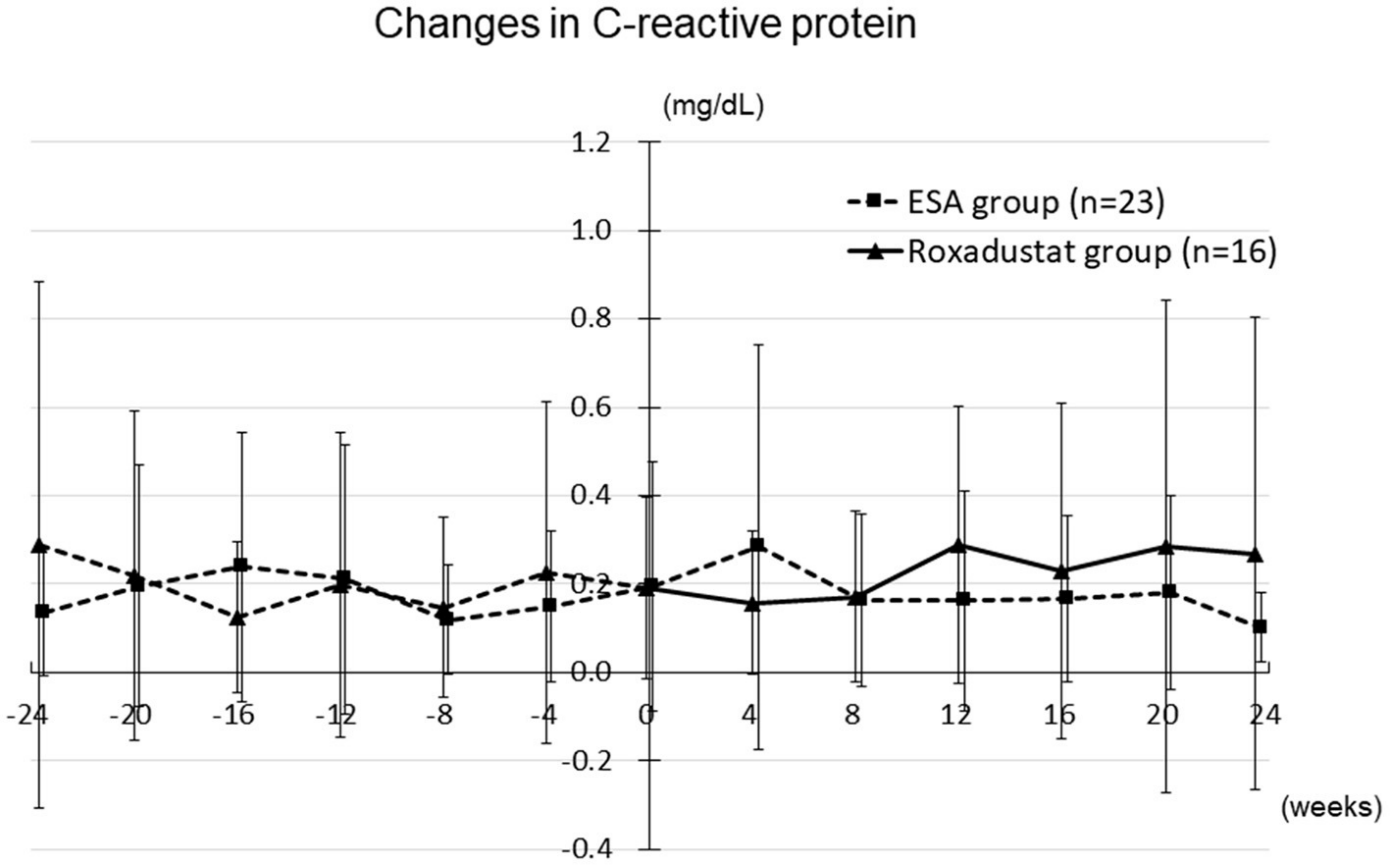
Changes in the C-reactive protein concentration in the Roxadustat and ESA groups. ESA, erythropoiesis-stimulating agent.

Other clinical and laboratory parameters, including body mass, systolic blood pressure, blood urea nitrogen, serum creatinine, uric acid, albumin, hemoglobin, sodium, potassium, chloride, calcium, phosphate, magnesium, intact-parathyroid hormone, and C-reactive protein (Figure 11) did not significantly differ from their baseline values in either the Roxadustat or ESA groups at 4, 8, 12, 16, 20, and 24 weeks (data not shown). An adverse effect was observed in one participant (nausea) in the Roxadustat group, and therefore the administration of roxadustat to this individual was discontinued.

## Discussion

In the present study, we found that roxadustat improved the anemia and reduced the serum ferritin concentration and TSAT of patients undergoing peritoneal dialysis after switching from ESA, without causing any serious adverse events. We also found that potassium concentration correlated with the dose of roxadustat. However, roxadustat did not affect the peritoneal membrane function or residual renal function of these patients.

Roxadustat is a hypoxia-inducible factor prolyl hydroxylase inhibitor that stabilizes hypoxia-inducible factor and stimulates erythropoietin gene expression. It increases the erythropoietin concentrations to within the physiological range in the kidneys and liver, which increases or maintains the hemoglobin concentration in patients with CKD and anemia (6). A recent clinical trial showed that roxadustat maintains the hemoglobin concentrations of patients undergoing peritoneal dialysis who are switched from an ESA (8). In this trial, the mean dose of roxadustat was not reduced and was 213.3 mg/week at 22 weeks in the ESA-Converted group. In the present study, the mean dose of roxadustat was almost unchanged and was 189 mg/week at 24 weeks, which is similar to the dose reported previously (8). In the present study, roxadustat increased the hemoglobin concentrations and stabilized them after switching from ESAs in a similar group of patients. It has been reported that the dose of ESA required to maintain the target hemoglobin concentration increased and the dose of roxadustat that was required decreased during the course of the randomized control trial that was conducted in hemodialysis patients (12). In the present study, the required dose of ESA continued to increase during the study period but the dose of roxadustat did not require to be increased after patients were switched from their ESA. These findings suggest that roxadustat improves and stabilizes hemoglobin concentrations without requiring any increase in dose in peritoneal dialysis patients with anemia that had previously been treated with ESA in a real-world clinical setting.

Serum ferritin and TSAT have been reported to increase in peritoneal dialysis patients with anemia who are treated with ESA as the duration of peritoneal dialysis increases (13), which has been attributed to the effects of pro-inflammatory cytokines, such as tumor necrosis factor-α and interleukin-1, and uremic toxins, including polyamines, as well as parathyroid hormone (14, 15). Roxadustat has been reported to improve iron metabolism through multiple pathways. It increases the gastrointestinal absorption of iron, promotes the release of stored iron from hepatocytes, and increases serum total iron-binding capacity (6). A recent phase III clinical study showed that roxadustat administration reduced serum ferritin and transferrin saturation after patients undergoing peritoneal dialysis were switched from an ESA (8). In the present study, serum ferritin and TSAT increased from baseline in the ESA group, but decreased in the Roxadustat group. As a result, the serum ferritin and transferrin saturation after baseline were lower in the Roxadustat group than in the ESA group. These results suggest that roxadustat may improve iron utilization in peritoneal dialysis patients with anemia who are being treated with an ESA in a real-world clinical setting.

It has been reported that several factors, including age, sex, and coexisting disease, affect the ESA dose requirement (16). In addition, ESA dose has been reported to correlate negatively with hemoglobin level (17). It has been reported that ESA dose-dependently increases the incidence of hypertension in patients with CKD (18). In contrast, the dose of roxadustat was not found to be associated with the hemoglobin concentration or systolic blood pressure in the present study. Furthermore, the dose of roxadustat was not related to indices of iron metabolism, such as ferritin and TSAT, or peritoneal dialysis-related parameters, including Kt/V and the duration of the peritoneal dialysis. These findings suggest that roxadustat is useful for the treatment of anemia in patients undergoing peritoneal dialysis and might be more efficacious than an ESA in patients who have anemia or hypertension. A phase III clinical study showed that roxadustat increases the risk of hyperkalemia in patients undergoing hemodialysis (12). In the present study, the dose of roxadustat positively correlated with the serum potassium concentration in patients undergoing peritoneal dialysis. These results suggest that roxadustat increases the risk of hyperkalemia in patients undergoing either hemodialysis or peritoneal dialysis. Therefore, physicians should be aware of the higher risk of hyperkalemia in patients with end-stage CKD who are taking roxadustat.

Roxadustat reportedly has several side effects including gastrointestinal disorders, nasopharyngitis, and back pain (7). In the present study, one patient in Roxadustat group had nausea. A phase III clinical trial involving peritoneal dialysis patients reported that gastrointestinal symptoms were observed in around 20% of the patients, which is compatible with our study findings (8). In the present study, roxadustat did not affect peritoneal membrane function or decline rate in residual renal function in peritoneal dialysis patients. These finding suggest that roxadustat might be safely used in patients undergoing peritoneal dialysis.

The present study had two main advantages over the previous study (8). First, it assessed the effects of roxadustat on peritoneal membrane function and residual renal function, and second, it identified the factors associated with the dose of roxadustat in patients undergoing peritoneal dialysis. Therefore, the results of the present study might be useful for further studies of the use of roxadustat in patients undergoing peritoneal dialysis.

The present study also had several limitations. First, it was a retrospective observational study; therefore, selection bias could not be completely eliminated and we might have underestimated the number of patients that experienced adverse events. Second, the study was performed at a single center, which limits the external validity of the results. Third, the number of participants in the study was small, which would have reduced the statistical power for the detection of differences between groups. Fourth, there was a lack of comparison of clinical features between two groups, which may have affected the results of the study. Fifth, we did not adjust for potential confounding factors when determining the effects of roxadustat, nor did we assess the long-term effects of roxadustat on peritoneal membrane function or residual renal function. Therefore, caution is needed when interpreting the study results including the association between the dose of roxadustat and serum potassium concentration. Further prospective, long-term, large, randomized clinical studies are required to investigate the effects of roxadustat on peritoneal membrane function and residual renal function and to identify the factors associated with the dose of roxadustat required to maintain optimal hemoglobin level in patients undergoing peritoneal dialysis.

In conclusion, roxadustat may improve the anemia and reduce the serum ferritin and TSAT of patients undergoing peritoneal dialysis, after they are switched from an ESA, without association with peritoneal membrane function or residual renal function. The results of the present study suggest that roxadustat is superior to ESAs with respect to its effects on anemia and iron metabolism in patients undergoing peritoneal dialysis.

## Data Availability Statement

The raw data supporting the conclusions of this article will be made available by the authors, without undue reservation.

## Ethics Statement

The studies involving human participants were reviewed and approved by Ethics Committee of Saitama Medical Center, Jichi Medical University. Written informed consent for participation was not required for this study in accordance with the national legislation and the institutional requirements.

## Author Contributions

TK and YMu conceived and designed the study. HN, MU, JM, SK, SM, YMu, MM, and HM collected the data. KY, HI, and AA analyzed the data. KH wrote the first draft of the manuscript. KI, YU, and SO made critical revisions. YMo approved the final version. All authors contributed to this manuscript and approved the final version for submission.

## Conflict of Interest

The authors declare that the research was conducted in the absence of any commercial or financial relationships that could be construed as a potential conflict of interest.
